# Real-world efficacy of MiniMed™780G recommended settings (glycemic target 100 mg/dL, active insulin time 2 hours) in youth and young adults with type 1 diabetes

**DOI:** 10.3389/fendo.2025.1670266

**Published:** 2025-09-12

**Authors:** Marta Bassi, Giordano Spacco, Federico Pezzotta, Margherita Di Jorgi, Giulia Siri, Andrea Pintabona, Maria Grazia Calevo, Nicola Minuto, Mohamad Maghnie

**Affiliations:** ^1^ DINOGMI - Department of Neuroscience, Rehabilitation, Ophthalmology, Genetics, Maternal and Child Health, University of Genoa, Genoa, Italy; ^2^ Pediatric Clinic, IRCCS Istituto Giannina Gaslini, Genoa, Italy; ^3^ Department of Pediatrics and Neonatology, IRCCS Istituto Giannina Gaslini, Pietra Ligure, Italy; ^4^ Clinical Psychology Unit, IRCCS Istituto Giannina Gaslini, Genoa, Italy; ^5^ Biostatistics Unit, Scientific Directorate, IRCCS Istituto Giannina Gaslini, Genoa, Italy

**Keywords:** AHCL (advanced hybrid closed loop), AID (automated insulin delivery), MiniMed 780G^®^, type 1 diabetes (T1D), CGM (continuous glucose monitoring), TIR (time in range), active insulin time (AIT), glucose target (GT)

## Abstract

**Background and aims:**

Despite growing evidence supporting the efficacy and safety of the MiniMed™ 780G recommended settings (Glucose Target 100 mg/dL and Active Insulin Time 2 hours), their adoption in routine practice remains limited, mainly due to concerns about hypoglycemia. This study aimed to evaluate the impact of switching to these settings in pediatric and young patients with type 1 diabetes (T1D).

**Methods:**

We conducted a retrospective longitudinal analysis in children and young adults using MiniMed™780G system at our center. Patients who switched from their initial settings to a glucose target of 100 mg/dL and an active insulin time of 2 hours for clinical indications were included. Data were retrospectively collected 3 months after switch. Glycemic metrics were compared over the 14 days before the switch (T0) and at 1 month (T1), and 3 months (T3).

**Results:**

Ninety-one patients with a mean age of 17.89y were included, 81.3% of whom already had a glucose target of 100 mg/dL at baseline. Therefore, in most cases the primary change was reducing AIT from 3 to 2 hours. After switching to the recommended settings, Time in Range (TIR) significantly increased (p<0.001) at T1 (71.9% *vs* 74.8%) and T3 (71.9% *vs* 75.0%). Time in target range (TITR) similarly improved from 47.2% at T0 to 51.4% at T1 and 50.9% at T3 (p<0.001) without any significant increase in time below range (TBR). The proportion of patients meeting all ADA-recommended glycemic targets rose from 29.5% at baseline to 40% at T3. Following the switch, the contribution of automatic correction boluses to the total insulin dose increased, while overall daily insulin requirements remained stable.

**Conclusions:**

Switching to the recommended MiniMed™780G settings, driven primarily by AIT reduction in most patients, was safe and effective, improving glycemic control without increasing hypoglycemia. These findings support broader use of these settings in pediatric and young adult patient with type 1 diabetes.

## 1 Introduction

Type 1 Diabetes (T1D) is one of the most common chronic diseases in childhood and adolescence and its incidence in Europe is steadily increasing (1). In recent years, the introduction of technological devices, particularly continuous glucose monitoring (CGM) sensors and insulin pumps with automatic insulin delivery (AID), has dramatically improved disease management. These tools optimize glycemic control and reduce the psychological burden on patients and families by decreasing the need for frequent therapeutic decisions (2–4).

AID systems can automatically adjust insulin delivery based on glycemic trends data received by the CGM (5). MiniMed™780G (Medtronic^®^, Northridge, California) is an Advanced Hybrid Closed Loop (AHCL) system that uses an interoperable Predictive Integrative Derivative – Insulin Feedback (PID-IFB) algorithm to deliver basal insulin and automatic correction boluses as needed. Basal insulin rates and insulin sensitivity factor (ISF), are largely based on insulin needs from the previous 5–7 days. The customizable parameters that allow the user to change the performance of the algorithm are the glycemic target (GT) and the active insulin time (AIT), in addition to the insulin to carbohydrates ratio (I/CHO). Users can adjust the glycemic target between 100, 110 and 120 mg/dl and an active insulin time from 2 to 8 hours (6, 7).

Several studies have demonstrated the positive impact of MiniMed™780G on glycemic outcomes. For instance, in a cohort of 101,629 users from 34 countries, the mean Time in Range 70–180 mg/dL (TIR) was 72,3%, with 62.5% of users achieving the recommended TIR > 70% (8, 9). MiniMed™780G proved to be effective also in the pediatric population, as showed by Castañeda et al., who reported a mean TIR of 71.2% and a mean Time in Tight Range 70–140 mg/dL (TITR) of 48.9% in 3762 users aged less than 15 years (10). Although not formally included in the technical specifications, a GT of 100 mg/dL and an AIT of 2 hours are commonly recommended settings, supported by in silico simulations, pilot studies conducted on early AHCL prototypes (11) and by early real-world evidence with the commercial MiniMed™780G system (12), to optimize glycemic outcomes.

Several studies have examined how pump settings influence glycemic outcomes, consistently finding that a GT of 100 mg/dl combined with an AIT of 2 hours is associated with the best glycemic control (8, 10, 12–15). However, none of these studies compared patients’ glycemic control before versus after implementing the recommended GT and AIT settings, leaving a gap in longitudinal evidence. Even though literature strongly supports the efficacy and safety of these settings, their uptake in clinical practice remains limited. Matejko et al. analyzed real-world data from the MiniMed™780G and reported that Polish patients achieved better glycemic control compared to the rest of Europe, correlating this result with a higher adherence to the manufacturer-recommended settings (16.9% *vs* 6.3%) (16). Nevertheless, a considerable proportion of clinicians across Europe continue to select higher glucose targets and longer AITs, likely due to persistent concerns about hypoglycemia, especially in pediatric patients. During our regular outpatient visits, starting from January 2024, we observed only a small subset of patients at our center have adopted the recommended settings.

Therefore, aligning with emerging literature supporting their efficacy and safety in youth, we offered the option to switch to MiniMed™780G users to GT 100 mg/dL and AIT 2 h when no hypoglycemia risk was present. The primary aim of our study was to evaluate, in a real-world retrospective setting, the impact of switching on glycemic control, specifically TIR, in pediatric and young adult users. Secondary aims included assessment of CGM metrics, insulin requirements, and algorithm-delivered insulin. To our knowledge, this is the first longitudinal analysis examining the same patients before and after switching to recommended settings.

## 2 Materials and methods

### 2.1 Study design and study population

In this single-center, retrospective, observational study, we analyzed real-world data to assess the performance, effectiveness and safety of adopting the MiniMed™780G recommended settings (GT 100 mg/dL – AIT 2 hours) in a cohort of pediatric and young adult patients with T1D. Glycemic and clinical data were compared at baseline (T0, when recommended settings were adopted), at 1 month (T1) and at 3 months (T3). The switch was offered during routine visits at the Pediatric Clinic, Endocrinology, Diabetes Center, of IRCCS Istituto Giannina Gaslini (Genoa, Italy) to patients meeting the following criteria: age over 7 years, use of the MiniMed™ 780G system for at least 1 month, no independent or clinically indicated changes to AIT and GT in the prior month, and no hypoglycemia concerns (TBR >10% or episodes of severe hypoglycemic in the past year).

For inclusion, patients needed to have adopted the recommended settings within the 12 months before data collection and maintained them unchanged throughout the study period, with at least 80% CGM and Automated Mode usage complied with the Helsinki Declaration and International Conference on Harmonization Good Clinical Practice guidelines, with anonymized data and informed consent from all patients or guardians. Ethics committee approval was not requested per Authorization no. 9/2014, which allows retrospective studies using coded data to proceed without additional ethical review.

### 2.2 Data collection

Data were extracted from the CareLink^®^ software for healthcare professionals. Data collection was performed considering the following times: T0 (14 days prior to the adoption of new settings of AIT 2h and GT 100 mg/dl), T1 (14 days prior to one month +/- 3 days from the start of the new parameters) and T3 (14 days prior to three months +/- 15 days from the start of the new parameters).

The primary outcome was the change in TIR from baseline after 1 and 3 months. Secondary outcomes included changes in other CGM metrics, such as TITR, Time Below Range 54–69 mg/dL (TBR), Time Below Range <54 mg/dL (TBR54), Time Above Range 181–250 mg/dL (TAR) and TAR > 250 mg/dL (TAR250), Glucose Management Indicator (GMI), Average Glucose (AG), Standard Deviation (SD) and Coefficient of Variation (CV). The percentage of CGM use and of time spent in Automated Mode were also investigated, as well as the daily insulin requirements (TDI, indicated in U/Kg/day).

Regarding insulin requirements, the following data were collected: total basal amount, total bolus amount at meals, auto correction bolus amount, average number of meals per day, average number of carbohydrates – CHO – per day relative to patient weight. In addition, the following data were collected for each patient at T0: demographic data (age, gender), medical history (age at onset of disease, duration of disease) and previous pump settings (AIT, GT). Episodes of DKA and severe hypoglycemia (SH) were also monitored during the 3- months study period.

### 2.3 Statistical analysis

Descriptive statistics were generated for the whole cohort. Data were expressed as mean and standard deviation for continuous variables and as absolute or relative frequencies for categorical variables. The distribution of the data was analyzed using the Kolmogorov–Smirnov test. Non-parametric statistics were considered as appropriate. Statistical analysis was carried out only on patients who used TG 100 mg/dL and 3 hours AIT at baseline (most frequently used settings) and who constituted the majority of cases. Comparisons between T0, T1 and T3 to examine continuous nonparametric variables were performed using Paired Wilcoxon test. P values ≤ 0.05 were considered statistically significant, and all P values were based on two tailed tests. Statistical analysis was performed using SPSS for Windows version 29 (SPSS Inc. Chicago, IL USA).

## 3 Results

### 3.1 Patients’ characteristics at baseline

Data from 91 MiniMed™780G users followed at the IRCCS Giannina Gaslini Pediatric Diabetes Center were analyzed. The mean age of the patients included in the study was 17.9 years (range 7.0–35.5), and 41.8% were female. At baseline, the most frequently used GT was 100 mg/dL (81.3%), while 9.9% and 8.8% of patients were using a GT of 110 mg/dL and 120 mg/dL, respectively. In contrast, only 5.5% of patients had an AIT of 2 hours at baseline, whereas an AIT of 3 hours was by far the most used (82.4%). Accordingly, the most frequent combination of settings at baseline was GT 100 mg/dL and AIT 3 hours, used by 70.3% of participants. The characteristics of the study population are summarized in **Table 1**.

**Table 1:** Patients’ characteristics at baseline Data are described as mean and standard deviation (SD) or median and range for continuous variables, and as absolute and relative frequencies for categorical variables.

Study population characteristics	Mean, median or frequency (total n=91)
Number of patients	91
Age (years)	17,89 ± 7,92
Sex assigned at birth	
Female	38 (41,8%)
Male	53 (58,2%)
GT - Glycemic Target (mg/dL)	
100	74 (81,3%)
110	9 (9,9%)
120	8 (8,8%)
AIT - Active Insulin Time (hours)	
2	5 (5,5%)
2,25	1 (1,1%)
2,5	5 (5,5%)
3	75 (82,4%)
4	4 (4,4%)
4,5	1 (1,1%)
Combination of GT 100 mg/dL and AIT 3 hours	64 (70.3%)
N. of DKA episodes in the previous year	0 (0%)
N. of SH episodes in the previous year	0 (0%)

### 3.2 Glycemic outcomes

**Figure 1:**
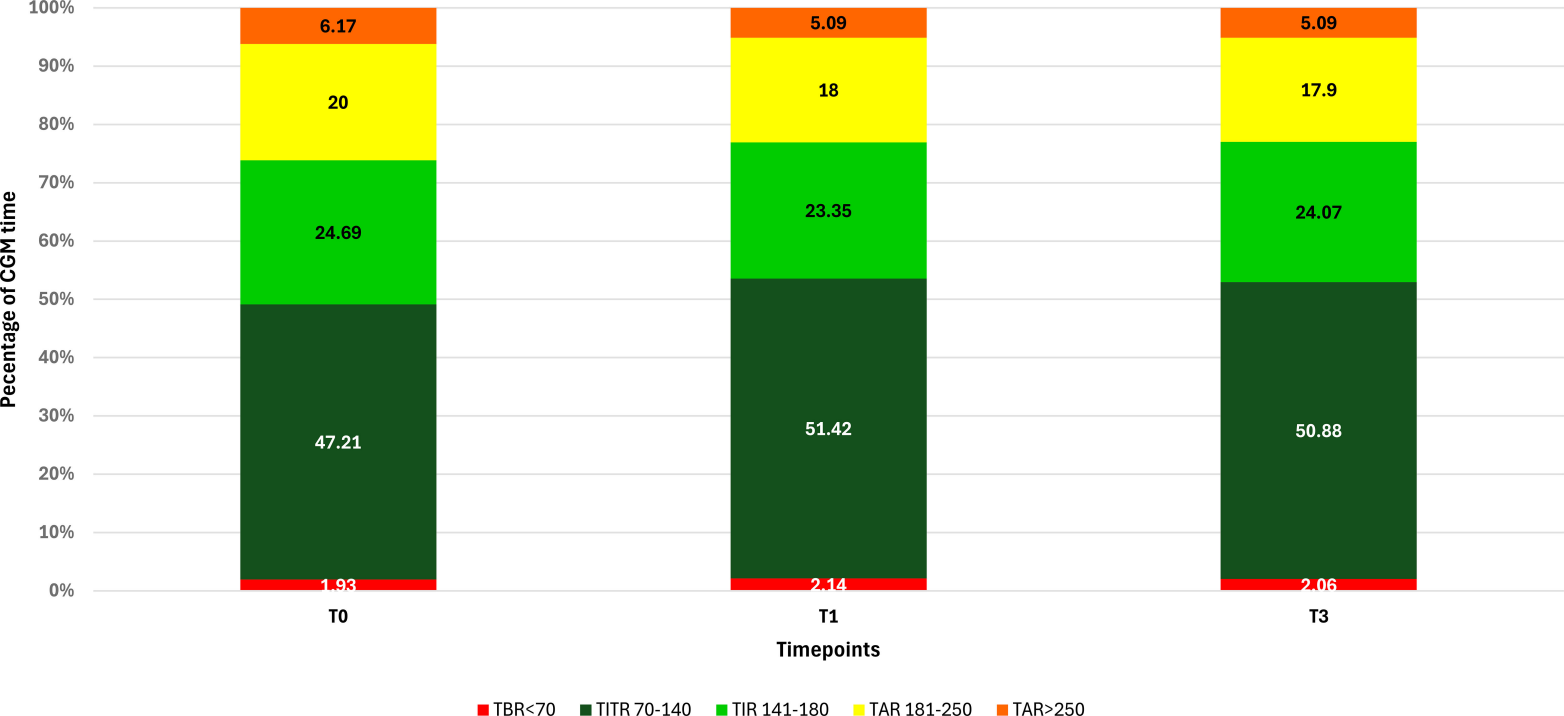
Changes in CGM metrics at baseline (T0) and after 1 (T1) and 3 months (T3) in the whole study population (N = 91).

**Figure 2:**
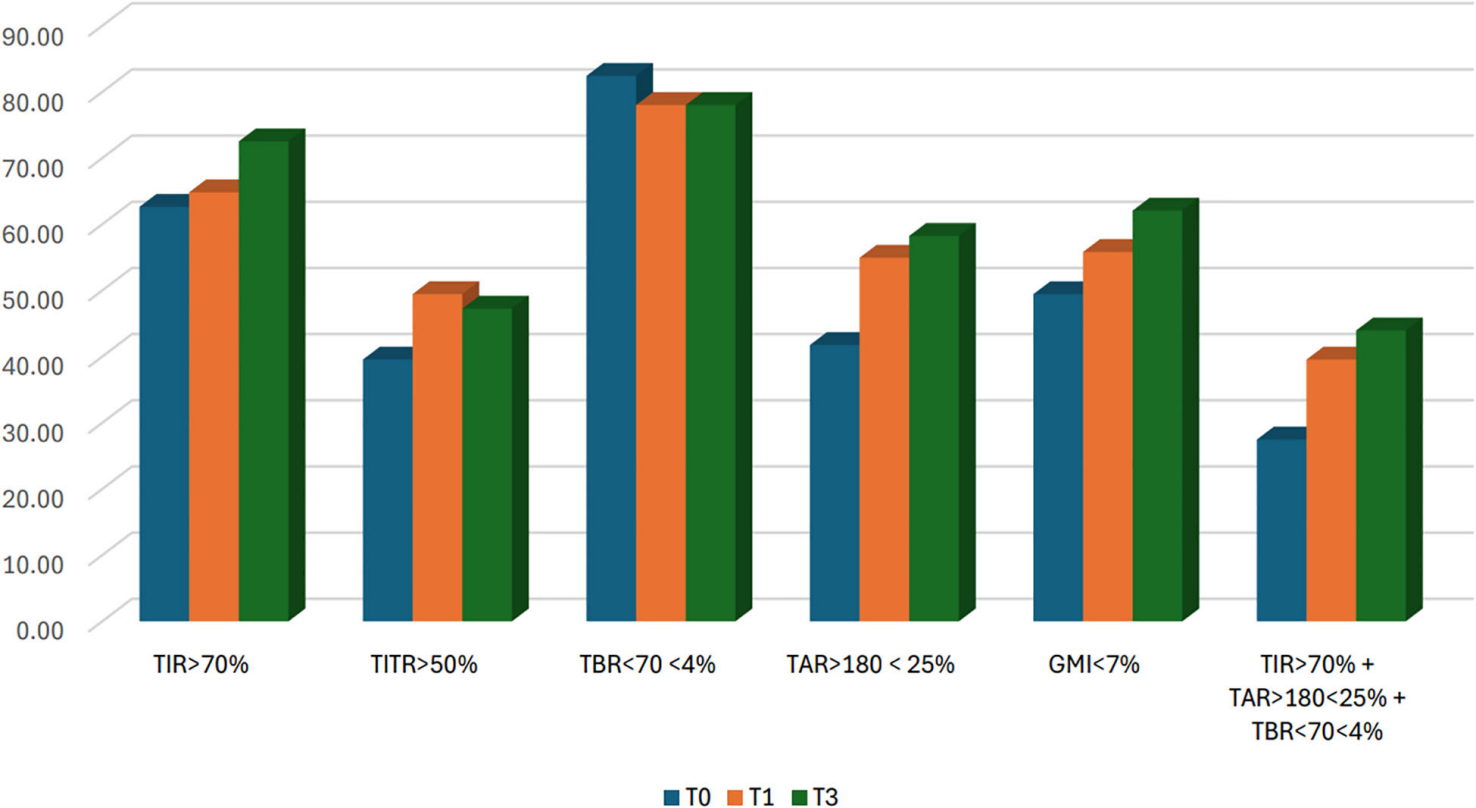
Percentage of patients meeting ADA recommended glycemic targets at baseline (T0) and after 1 (T1) and 3 months (T3). N = 91 (in 2 patients GMI was not available).

A significant increase in TIR (p < 0.001) was observed both at T1 (71.90% *vs* 74.77%; +2.9%) and T3 (71.90% *vs* 74.95%; +3.0%), along with an improvement in TITR (p < 0.001) at both time points (47.21% *vs* 51.42%; +4.2% at T1 and 47.21% *vs* 50.88%; +3.7% at T3). These improvements were associated, both at T1 and T3, with a significant reduction in TAR (p < 0.001) and no significant increase in time spent in hypoglycemia. Average glucose and GMI also showed a significant reduction (p < 0.001) at both T1 and T3. Some secondary metrics, such as TAR250 and SD, although significantly reduced at T1, did not maintain statistical significance at T3. (**Figure 1**) Data and comparisons of CGM metrics across the different time points are presented in **Table 2**. The switch to the recommended settings increased the percentage of patients achieving the ADA-recommended glycemic targets (17): at T3, 72.5% of patients achieved a TIR >70% compared to 62.6% at baseline, and 47.2% reached a TITR >50% compared to 39.6% at baseline. The proportion of patients meeting all glycemic targets (TIR >70%, TAR >180 mg/dL <25%, and TBR <70 mg/dL <4%) increased from 29.5% at baseline to 40% at T3 (**Figure 2**). Regarding safety, no episodes of DKA or SH were reported during the study period. Given that the majority of patients included were using a GT of 100 mg/dL and an AIT of 3 hours at baseline (N = 64; 70.3%), a sub-analysis was conducted specifically in this subgroup. The findings were consistent with those observed in the entire cohort: a significant increase in TIR was seen at both T1 (72.16% *vs* 74.55%; +2.3%, p = 0.02) and T3 (72.16% *vs* 74.34%; +2.1%, p = 0.03), and TITR also improved at both time points (47.64% *vs* 51.31%; +3.7%, p = 0.003 at T1 and 47.64% *vs* 50.56%; +3.0%, p = 0.01 at T3), with a significant reduction in TAR and no significant increase in TBR. The results of the sub-analysis are presented in **Table 3**. A subgroup analysis by age (<18 *vs* ≥18 years) was also performed; results are presented in **Supplementary Tables 1, 2**.

**Table 2:** Metrics of all patients (N = 91) at baseline (T0) and after 1 (T1) and 3 months (T3) from baseline.

CGM metrics and insulin delivery	T0	T1	P *(T1 vs T0)*	T3	P *(T3 vs T0)*
**TIR%**	71,90 ± 9,94	74,77 ± 9,19	**0,001**	74,95 ± 9,52	**<0,001**
**TITR%**	47,21 ± 10,22	51,42 ± 11,24	**<0,001**	50,88 ± 9,95	**<0,001**
**TAR%**	20,32 ± 5,87	17,98 ± 6,36	**<0,001**	17,89 ± 5,82	**<0,001**
**TAR250%**	6,19 ± 6,20	5,09 ± 4,45	**0,04**	5,09 ± 4,69	0,06
**TBR%**	1,63 ± 1,50	1,82 ± 1,76	0,29	1,71 ± 1,55	0,82
**TBR54%**	0,30 ± 0,55	0,32 ± 0,53	0,69	0,35 ± 0,64	0,38
**AG (mg/dl)**	152,79 ± 17,03	147,51 ± 16,09	**0,001**	147,95 ± 14,46	**0,001**
**SD (mg/dl)**	52,62 ± 10,50	50,58 ± 10,70	**0,02**	51,15 ± 11,39	0,15
**GMI (%)**	6,95 ± 0,36	6,85 ± 0,38	**0,003**	6,84 ± 0,36	**0,001**
**CV (%)**	34,32 ± 4,68	34,15 ± 5,14	0,85	34,31 ± 5,14	0,96
**TDI (U/kg/day)**	0,79 ± 0,28	0,79 ± 0,25	0,48	0,79 ± 0,26	0,16
**Total Bolus (U/kg/day)**	0,45 ± 0,18	0,47 ± 0,17	**0,005**	0,48 ± 0,17	**0,003**
**Auto Correction Bolus (U/kg/day)**	0,14 ± 0,08	0,17 ± 0,09	**<0,001**	0,18 ± 0,09	**<0,001**
**Meal Bolus (U/kg/day)**	0,32 ± 0,14	0,30 ± 0,12	**0,05**	0,30 ± 0,11	0,50
**Basal (U/kg/day)**	0,35 ± 0,19	0,32 ± 0,10	**0,01**	0,32 ± 0,11	0,33
**Meals (n/day)**	4,15 ± 1,35	3,82 ± 1,35	**0,004**	3,87 ± 1,14	0,16
**CHO (g/day)**	196,78 ± 65,34	186,68 ± 77,76	**0,006**	185,48 ± 65,84	0,12
**CHO/kg (g/Kg/day)**	3,94 ± 1,94	3,70 ± 2,00	**0,004**	3,66 ± 1,42	**0,05**

Data are expressed as mean ± SD. Bold, statistically significant.

TIR, Time in Range 70–140 mg/dL; TITR, Time in Tight range 70–140 mg/dL; TAR, Time Above Range 181–250 mg/dL; TAR250, Time Above Range > 250 mg/dL; TBR, Time Below Range 54–69 mg/dL; TBR54, Time Below Range < 54 mg/dL AG, Average Glucose; SD, Standard Deviation; GMI, Glucose management Indicator; CV, Coefficient of Variation; TDI, Total Daily Insulin Requirement; CHO, carbohydrates.

**Table 3:** Metrics of patients using at baseline a GT of 100 mg/dL and an AIT of 3 hours (n= 64); at T0 and after 1 (T1) and 3 months (T3) from baseline.

CGM metrics and insulin delivery	T0	T1	P *(T1 vs T0)*	T3	P *(T3 vs T0)*
**TIR%**	72,16 ± 10,83	74,55 ± 9,60	**0,02**	74,34 ± 10,29	**0,03**
**TITR%**	47,64 ± 10,30	51,31 ± 11,03	**0,003**	50,56 ± 10,08	**0,01**
**TAR%**	19,95 ± 6,03	18,22 ± 5,90	**0,006**	18,17 ± 5,76	**0,008**
**TAR250%**	6,42 ± 6,92	5,38 ± 4,76	0,10	5,48 ± 5,29	0,27
**TBR%**	1,52 ± 1,39	1,59 ± 1,42	0,64	1,64 ± 1,47	0,52
**TBR54%**	0,27 ± 0,51	0,23 ± 0,46	0,62	0,34 ± 0,62	0,27
**AG (mg/dl)**	153,06 ± 18,15	148,48 ± 15,46	**0,02**	149,03 ± 15,08	**0,05**
**SD (mg/dl)**	52,72 ± 11,81	51,08 ± 11,63	0,08	51,67 ± 12,81	0,45
**GMI (%)**	6,95 ± 0,38	6,88 ± 0,36	0,07	6,87 ± 0,37	**0,04**
**CV (%)**	34,26 ± 5,11	34,16 ± 5,46	0,99	34,25 ± 5,43	0,97
**TDI (U/kg/day)**	0,79 ± 0,25	0,79 ± 0,23	0,66	0,78 ± 0,26	0,21
**Total Bolus (U/kg/day)**	0,44 ± 0,16	0,47 ± 0,16	**0,002**	0,47 ± 0,16	**0,005**
**Auto Correction Bolus (U/kg/day)**	0,14 ± 0,07	0,18 ± 0,10	**0,001**	0,18 ± 0,10	**<0,001**
**Meal Bolus (U/kg/day)**	0,32 ± 0,13	0,29 ± 0,10	0,08	0,28 ± 0,11	0,35
**Total Basal (U/kg/day)**	0,37 ± 0,21	0,32 ± 0,09	**0,003**	0,29 ± 0,10	0,20
**Meals (n/day)**	4,13 ± 1,34	3,73 ± 1,26	**0,004**	3,86 ± 1,36	0,27
**CHO (g/day)**	199,38 ± 68,63	185,02 ± 79,01	**0,003**	189,20 ± 61,90	0,28
**CHO/kg (g/Kg/day)**	3,88 ± 1,89	3,73 ± 1,26	**0,002**	3,59± 1,87	0,12

Data are expressed as mean ± SD. Bold, statistically significant. TIR, Time in Range 70–140 mg/dL; TITR, Time in Tight range 70–140 mg/dL; TAR, Time Above Range 181–250 mg/dL; TAR250, Time Above Range > 250 mg/dL; TBR, Time Below Range 54–69 mg/dL; TBR54, Time Below Range < 54 mg/dL AG, Average Glucose; SD, Standard Deviation; GMI, Glucose management Indicator; CV, Coefficient of Variation; TDI, Total Daily Insulin Requirement; CHO, carbohydrates.

### 3.3 Insulin delivery

In the overall study population (**Table 2**), the total daily insulin requirement (TDI) did not show any statistically significant change at either T1 or T3. However, the distribution of insulin delivery changed significantly over time: the total amount of bolus insulin increased at both time points, entirely due to the significant rise in auto-correction boluses, which increased from 0.14 U/kg/day at baseline to 0.17 U/Kg/day at T1 and 0.18 U/Kg/day at T3 (p < 0.001), while manually delivered meal boluses showed a significant decrease at T1 (p=0.05). Basal insulin delivery also showed a slight but significant reduction at T1 (p = 0.01). Patient interaction with the pump decreased over the study period, as demonstrated by a significant reduction at T1 in the number of meals entered (from 4.15 to 3.82 meals/day) and the amount of carbohydrates declared (from 3.94 to 3.70 g/kg/day). Findings from the subgroup of patients with GT 100 mg/dL and AIT 3 hours at baseline were consistent with those observed in the entire population (**Table 3**).

## 4 Discussion

In recent years, multiple studies have ed confirmed that Medtronic’s recommended MiniMed™ 780G settings, AIT of 2 hours and GT of 100 mg/dL, consistently deliver superior glycemic control compared to other configurations. However, to date, no research has evaluated the impact of switching to these settings longitudinally within the same patients, rather than comparing different settings in distinct patients.

In 2022, Arrieta et al. (13) analyzed data from 12,870 MiniMed™780G users, including 3,211 pediatric patients (<15 years). Pediatric users of GT 100 mg/dL and AIT 2 hours achieved a higher mean TIR (78.9%) than the overall pediatric group (TIR 73.9%), with an average TBR <70 mg/dL of 3.6%. Those using GT 110 mg/dL with AIT 2 hours had slightly lower TIR (76.5%) but lower TBR (2.7%), suggesting starting with GT 110 mg/dL may be prudent in younger patients, then reducing to 100 mg/dL if hypoglycemia risk is low. Castañeda et al., using the same dataset, identified a GT of 100mg/dl and an AIT of 2 hours as the strongest predictor of increased TIR (mean 80.7%) with low TBR (3.0%). Shortening AIT further improved TIR without affecting TBR, reinforcing its safety (18).

A larger real-world analysis of 101,629 including 22,541 pediatric patients (<15 years) by Choudhary et al. across 34 countries, showed that optimal setting achieved mean TIR of 76.2% *vs* 69.9% (8).

The only available prospective trial assessing the impact of adjusting glucose target and AIT was conducted on 157 individuals, including 39 adolescents (aged 14–21 years), using a prototype AHCL algorithm implemented on the MiniMed™670G version 4.0. In this single-arm study, Carlson et al. reported an increase in TIR from 68.8% during the run-in period to 74.5% after initiating AHCL. Subsequently, lowering the glucose target to 100 mg/dL and reducing the AIT to 2 hours further improved TIR to 75.4%, with no increase in TBR (19).

Better glycemic control with these optimized settings has also been demonstrated for the more stringent Time in Tight Range (TITR). In a cohort of 111 children and adolescents, Bombaci et al. found that patients who achieved a TITR ≥ 50% with TBR ≤ 4% were significantly more likely to be using the optimal settings (AIT of 2 hours and a GT of 100 mg/dL) compared to those who did not (14). Trasher et al. further confirmed this evidence in a large real-world analysis of 7,499 MiniMed™ 780G users in the USA. They reported an overall mean TITR of 51.4%, which increased to 56.4% among users with the recommended GT and AIT settings. Notably, more than 80% of these users met the recommended goal for each individual metric, and over 75% achieved combined goals for GMI, TIR, TAR, and TBR (15). An even larger real-world dataset of 13,461 users, including 3,762 pediatric patients, showed that among those consistently using GT 100 mg/dL and AIT 2 h, mean TITR was 56.7–57.0%. They also demonstrated that the impact of GT and AIT on TITR was approximately 60% greater than on TIR, supporting the use of TITR >50% as a complementary therapeutic target (10). Finally, Matejko et al. analyzed data from 1,304 Polish MiniMed™780G users with 55,659 users from other European countries. The Polish cohort achieved a significantly higher mean TIR (79.1% *vs* 73.0%), which was largely attributed to the greater adoption of the optimal settings (19.7% in Poland *vs* 6.3% in the rest of Europe) (16). These findings reinforce that, despite substantial evidence supporting safety and efficacy, the recommended parameters remain underutilized in clinical practice, likely due to persistence of unfounded concerns regarding hypoglycemia risk.

Consistent with broader adoption patterns, only a minority of patients in our center utilized the recommended GT and AIT settings. Recognizing this, we systematically adjusted these parameters during routine follow-ups and then performed a retrospective analysis to assess the real-world impact of our approach.

Our findings strengthen previously published evidence demonstrating that patients on optimal settings generally achieve better glycemic outcomes. In our study, most participants (81.3%) were already using the 100 mg/dL glucose target at baseline, so the observed improvements are likely largely attributable to the reduction of AIT from 3 to 2 hours. Specifically, we observed an increase in TIR of 2.87% at T1 and 3.05% at T3, corresponding to approximately 44 additional minutes per day at T3 spent within the target range. Even modest increases in TIR have been associated with a meaningful reduction in the risk of microvascular and cardiovascular complications, as highlighted by the 2019 international consensus on TIR, underscoring the clinical importance of the improvements observed in our cohort (20). The improvement in glycemic control we observed was entirely driven by an increase in TITR, which rose by 4.21% at T1 and 3.68% at T3, corresponding to approximately 53 additional minutes per day within the 70–140 mg/dL range at T3.

Notably, switching to the optimal settings produced a relative effect on TITR that was approximately 88% greater than for TIR, underscoring the greater sensitivity of TITR as an additional glycemic metric. This implies that the observed gain in TIR reflects a genuine shift towards tighter and safer glucose control and not just fewer hyperglycemic excursions. Moreover, the proportion of patients in our cohort who met all ADA-recommended glycemic targets (TIR>70%, TAR >180 mg/dL <25%, TBR <70 mg/dL <4%) increased from 29.5% at baseline to 40% at T3. Importantly, this improvement was achieved without any significant increase in hypoglycemia, and no episodes of severe hypoglycemia occurred, addressing key concerns that often limits the adoption of more aggressive AHCL settings. While TIR and TITR improved and TAR decreased, a key indicator of glycemic variability, such as CV, remained stable across timepoints. This may reflect that reducing AIT enhanced the system’s ability to blunt hyperglycemia, lowering the amplitude of highs, but without substantially altering variability patterns, which are often driven by suboptimal pre-meal bolus timing and dosing.

These results, derived from longitudinal follow-up of the same pediatric and young adult cohort, further affirm that adopting the recommended settings is both effective and safe in real-world conditions. We also examined the largest subgroup of our patients already using GT of 100 mg/dL but with AIT of 3 hours at baseline, comprising 70.3% of our study cohort highlighting that the primary intervention was AIT reduction. Within this subgroup, similar improvements were observed: TIR increased by 2.39% at T1 and 2.18% at T3, while TITR rose by 3.67% at T1 and 3.08% at T3. Concurrently, TAR decreased and TBR remained stable, with no significant increase in hypoglycemia. An additional age-stratified sub-analysis (<18 years *vs* > 18 years) was performed. Adults showed a slightly greater improvement in TIR and TITR compared with pediatric participants, without differences in prandial insulin delivery or carbohydrate entries. This suggests that the larger effect in adults was likely due to their lower baseline glycemic control rather than to differences in system use.

These findings underscore the pivotal role of adopting a lower AIT in enhancing glycemic control. Due to the high baseline homogeneity, direct stratification by GT versus AIT adjustments was not feasible. However, our subgroup analysis focusing on patients whose primary change was reducing AIT clearly indicates that the observed improvements, particularly the stability of TBR, were predominantly driven by AIT reduction, consistent with Castañeda et al. (18).

Regarding insulin delivery, our data demonstrate that the total daily insulin dose remained stable, but its distribution shifted. In particular, the automatic correction boluses increased significantly, especially between T0 and T1, while manual meal boluses decreased slightly, and basal insulin showed a modest reduction. These findings reflect a greater reliance on the system’s automated adjustments, particularly autocorrections, when recommended settings are adopted.

Finally, the observed benefits, including the increase in TIR and, even more importantly, the improvement in TITR reaching the recommended targets, underscore the importance of adopting the Medtronic^®^ recommended settings. While it may be prudent to start MiniMed™780G users, especially very young children, on more conservative parameters to mitigate hypoglycemia risk, our findings suggest that once this risk is effectively managed or ruled out, clinicians should gradually optimize settings toward the recommended values to maximize glycemic outcomes. This study has both strengths and limitations. Among its strengths, this is the first real-world longitudinal evaluation of the impact of switching to recommended MiniMed™780G settings on glycemic outcomes within the same cohort over time. Our data demonstrate that this optimization is safe and effective in children and young adults, with no increase in hypoglycemia.

Notably, nearly all patients achieved glycemic targets during a critical stage of life for brain and cognitive development as well as for the prevention of long-term complications. This study provides valuable real-world data from a relatively large cohort, helping to inform how best to optimize system use in everyday clinical practice. Additionally, we evaluated the impact not only on TIR but also on TITR, a promising and more stringent CGM metric that may offer further insight into glycemic optimization. Indeed, this study has both strengths and limitations. Among its strengths, it represents the first real-world longitudinal evaluation of the impact of switching to the recommended MiniMed™780G settings on glycemic outcomes within the same cohort over time. Our data demonstrate that this optimization is both safe and effective in children and young adults, with no observed increase in hypoglycemia. However, certain limitations must be acknowledged: the retrospective design and the absence of a control group limit our ability to directly compare outcomes across different settings. A control group formed by non-switchers was not included because these patients had clear clinical contraindications (e.g., age <7 years, major hypoglycemia concerns), and including them would have introduced substantial confounding by indication. The follow-up period was relatively short, and only a small proportion of patients changed their glucose target, which restricts the generalizability of conclusions regarding the efficacy and safety of lowering the GT specifically. Furthermore, the high proportion of patients already using the recommended GT at baseline prevented stratified analyses that could disentangle the independent effects of GT and AIT changes. Future prospective studies with longer follow-up periods and more variability in baseline settings are needed to confirm and extend these findings.

## 5 Conclusions and future perspectives

This study is the first real-world, longitudinal evaluation to demonstrate the safety and effectiveness of switching to the Medtronic^®^ recommended MiniMed™ 780G settings (AIT of 2 hours and GT of 100 mg/dL) in children and young adults with T1D. Reducing AIT from 3 to 2 hours emerged as a key driver of improved glycemic control, resulting in a meaningful increase in both TIR and TITR while maintaining stable hypoglycemia rates. These results reinforce the clinical value of TITR as a sensitive and complementary metric for assessing glycemic optimization. Moreover, the proportion of patients achieving all ADA-recommended glycemic targets increased substantially, and no episodes of severe hypoglycemia were reported. These improvements were observed without changes in total insulin dose, highlighting a shift toward increased reliance on automated system functions, such as correction boluses.

Despite supporting evidence, adoption of the recommended settings remains limited in clinical practice, likely due to persistent concerns about hypoglycemia. Our data challenge these concerns and support a more proactive approach to optimizing pump settings, particularly after initial safety has been established. Further prospective studies with longer follow-up, greater diversity in age and baseline settings, and the inclusion of children under 7 years are warranted to validate these findings and guide broader implementation of optimal MiniMed™780G settings in routine diabetes care.

## Data Availability

The original contributions presented in the study are included in the article/**Supplementary Material**. Further inquiries can be directed to the corresponding author.

## References

[1] Ruiz-Grao MC Díez-Fernández A Mesas AE Martínez-Vizcaíno V Sequí-Domínguez I Sebastián-Valles F. Trends in the incidence of type 1 diabetes in European children and adolescents from 1994 to 2022: A systematic review and meta-analysis. Pediatr Diabetes. (2024) 2024:2338922. doi: 10.1155/2024/2338922, PMID: 40302967 PMC12020782

[2] Teo E Hassan N Tam W Koh S. Effectiveness of continuous glucose monitoring in maintaining glycaemic control among people with type 1 diabetes mellitus: a systematic review of randomised controlled trials and meta-analysis. Diabetologia. (2022) 65:604–19. doi: 10.1007/s00125-021-05648-4, PMID: 35141761

[3] Enes Romero P Güemes M Guijo B Martos-Moreno GÁ Pozo Román J Argente J. Automated insulin delivery systems in the treatment of diabetes: Benefits, challenges, and practical considerations in pediatric patients. Endocrinol Diabetes Nutr (Engl Ed). (2024) 71:436–46. doi: 10.1016/j.endien.2024.11.010, PMID: 39567321

[4] Yang Q Zeng B Hao J Yang Q Sun F. Real-world glycaemic outcomes of automated insulin delivery in type 1 diabetes: A meta-analysis. Diabetes Obes Metab. (2024) 26:3753–63. doi: 10.1111/dom.15718, PMID: 38888056

[5] Adolfsson P Hanas R Zaharieva DP Dovc K Jendle J. Automated insulin delivery systems in pediatric type 1 diabetes: A narrative review. J Diabetes Sci Technol. (2024) 18:1324–33. doi: 10.1177/19322968241248404, PMID: 38785359 PMC11535396

[6] Grosman B Roy A Lintereur L Turksoy K Benedetti A Cordero TL. A peek under the hood: explaining the miniMed™ 780G algorithm with meal detection technology. Diabetes Technol Ther. (2024) 26:17–23. doi: 10.1089/dia.2023.0446, PMID: 38377324

[7] Bassi M Franzone D Dufour F Strati MF Scalas M Tantari G. Automated insulin delivery (AID) systems: use and efficacy in children and adults with type 1 diabetes and other forms of diabetes in europe in early 2023. Life (Basel). (2023) 13:783. doi: 10.3390/life13030783, PMID: 36983941 PMC10053516

[8] Choudhary P Arrieta A van den Heuvel T Castanñeda J Smaniotto V Cohen O. Celebrating the data from 100,000 real-world users of the miniMed™ 780G system in Europe, Middle East, and Africa collected over 3 years: from data to clinical evidence. Diabetes Technol Ther. (2024) 26:32–7. doi: 10.1089/dia.2023.0433, PMID: 38377326 PMC10890936

[9] de Bock M Agwu JC Deabreu M Dovc K Maahs DM Marcovecchio ML. International society for pediatric and adolescent diabetes clinical practice consensus guidelines 2024: glycemic targets. Horm Res Paediatr. (2024) 97:546–54. doi: 10.1159/000543266, PMID: 39701064 PMC11854972

[10] Castanñeda J Arrieta A van den Heuvel T Battelino T Cohen O. Time in tight glucose range in type 1 diabetes: predictive factors and achievable targets in real-world users of the miniMed 780G system. Diabetes Care. (2024) 47:790–7. doi: 10.2337/dc23-1581, PMID: 38113453 PMC11043222

[11] Collyns OJ Meier RA Betts ZL Chan DSH Frampton C Frewen CM. Improved glycemic outcomes with medtronic miniMed advanced hybrid closed-loop delivery: results from a randomized crossover trial comparing automated insulin delivery with predictive low glucose suspend in people with type 1 diabetes. Diabetes Care. (2021) 44:969–75. doi: 10.2337/dc20-2250, PMID: 33579715

[12] Silva JD Lepore G Battelino T Arrieta A Castanñeda J Grossman B. Real-world performance of the miniMed™ 780G system: first report of outcomes from 4120 users. Diabetes Technol Ther. (2022) 24:113–9. doi: 10.1089/dia.2021.0203, PMID: 34524003 PMC8817690

[13] Arrieta A Battelino T Scaramuzza AE Da Silva J Castanñeda J Cordero TL. Comparison of MiniMed 780G system performance in users aged younger and older than 15 years: Evidence from 12 870 real-world users. Diabetes Obes Metab. (2022) 24:1370–9. doi: 10.1111/dom.14714, PMID: 35403792 PMC9545031

[14] Bombaci B Passanisi S Calderone M Macrì F Lombardo F Salzano G. Long-term use of Minimed™ 780G in children and adolescents with type 1 diabetes under real-world conditions: The benefits of optimal settings. Diabetes Obes Metab. (2025) 27:2309–12. doi: 10.1111/dom.16226, PMID: 39887516 PMC11885100

[15] Thrasher JR Arrieta A Niu F Cameron KR Cordero TL Shin J. Early real-world performance of the miniMed™ 780G advanced hybrid closed-loop system and recommended settings use in the United States. Diabetes Technol Ther. (2024) 26:24–31. doi: 10.1089/dia.2023.0453, PMID: 38377317

[16] Matejko B van den Heuvel T Castaneda J Arrieta A Cyranka K Cohen O. Excellence in the management of Advanced Hybrid Closed-Loop Systems: Lessons from the Polish cohort. Diabetes Res Clin Pract. (2024) 216:111832. doi: 10.1016/j.diabres.2024.111832, PMID: 39173678

[17] American Diabetes Association Professional Practice Committee. 6. Glycemic goals and hypoglycemia: standards of care in diabetes-2024. Diabetes Care. (2024) 47:S111–25. doi: 10.2337/dc24-S006, PMID: 38078586 PMC10725808

[18] Castanñeda J Mathieu C Aanstoot HJ Arrieta A Da Silva J Shin J. Predictors of time in target glucose range in real-world users of the MiniMed 780G system. Diabetes Obes Metab. (2022) 24:2212–21. doi: 10.1111/dom.14807, PMID: 35791621

[19] Carlson AL Sherr JL Shulman DI Garg SK Pop-Busui R Bode BW. Safety and glycemic outcomes during the miniMed™ Advanced hybrid closed-loop system pivotal trial in adolescents and adults with type 1 diabetes. Diabetes Technol Ther. (2022) 24:178–89. doi: 10.1089/dia.2021.0319, PMID: 34694909 PMC8971997

[20] Battelino T Danne T Bergenstal RM Amiel SA Beck R Biester T. Clinical targets for continuous glucose monitoring data interpretation: recommendations from the international consensus on time in range. Diabetes Care. (2019) 42:1593–603. doi: 10.2337/dci19-0028, PMID: 31177185 PMC6973648

