# Supplementary material for: Real-world efficacy of MiniMed™780G recommended settings (glycemic target 100 mg/dL, active insulin time 2 hours) in youth and young adults with type 1 diabetes
Source: Front Endocrinol (Lausanne). 2025 Sep 12;16:1670266. doi: 10.3389/fendo.2025.1670266 (PMC12463605; doi:10.3389/fendo.2025.1670266)
Supplement: Supplementary file 1 [file Table1.docx]

**Supplementary Table 1**. Metrics of all patients (N=91) at baseline (T0) and after 1 (T1) and 3 months (T3) from baseline. Data are expressed as mean ± SD. Bold = statistically significant. **<18 yrs n=59**

|  | **T0** | **T1** | **P**  ***(T1 vs T0)*** | **T3** | **P**  ***(T3 vs T0)*** |
| --- | --- | --- | --- | --- | --- |
| **TIR%** | 73,22 ± 9,82 | **75,95 ± 9,47** | **0,006** | 75,36 ± 9,92 | **0,02** |
| **TITR%** | **48,73± 10,22** | **52,42 ± 11,71** | **0,002** | 51,90 ± 10,14 | **0,007** |
| **TAR%** | **19,17 ± 5,71** | **17,36 ± 6,35** | **0,003** | 17,37 ± 5,79 | **0,007** |
| **TAR250%** | **5,73 ± 6,07** | **4,51 ± 4,37** | **0,02** | 4,85 ± 4,84 | 0,18 |
| **TBR%** | **1,88 ± 1,60** | **1,85 ± 1,60** | 0,81 | 1,98 ± 1,62 | 0,72 |
| **TBR54%** | **0,34 ± 0,58** | **0,34 ± 0,54** | 1 | 0,42 ± 0,67 | 0,32 |
| **AG (mg/dl)** | **150,15 ± 16,58** | **145,44 ± 15,97** | **0,01** | 146,19 ± 14,85 | **0,03** |
| **SD (mg/dl)** | **52,19 ± 10,88** | **49,08 ± 10,98** | **0,003** | 51,03 ± 11,82 | 0,43 |
| **GMI (%)** | **6,89 ± 0,36** | **6,80 ± 0,38** | **0,04** | 6,80 ± 0,36 | **0,03** |
| **CV (%)** | **34,61 ± 5,04** | **33,59 ± 5,45** | 0,15 | 34,52 ± 5,17 | 0,84 |
| **TDI (U/kg/day)** | **0,80 ± 0,29** | **0,80 ± 0,24** | 0,87 | 0,82 ± 0,27 | **0,02** |
| **Total Bolus (U/kg/day)** | **0,46 ± 0,18** | **0,48 ± 0,16** | **0,01** | 0,50 ± 0,16 | **0,001** |
| **Auto Correction Bolus (U/kg/day)** | **0,14 ± 0,07** | **0,17 ± 0,09** | **<0,001** | 0,18 ± 0,09 | **<0,001** |
| **Meal Bolus (U/kg/day)** | **0,32 ± 0,13** | **0,31 ± 0,11** | 0,18 | 0,32 ± 0,11 | 0,97 |
| **Basal (U/kg/day)** | **0,34 ± 0,13** | **0,32 ± 0,10** | **0,01** | 0,33 ± 0,12 | 0,81 |
| **Meals (n/day)** | **4,40 ± 1,34** | **3,99 ± 1,30** | **0,003** | 4,11 ± 1,50 | 0,32 |
| **CHO (g/day)** | **203,40 ± 63,24** | **195,65 ± 78,78** | **0,04** | 195,84 ± 63,26 | 0,68 |
| **CHO/kg (g/Kg/day)** | **4,38 ± 2,05** | **4,15 ± 1,98** | **0,03** | 4.17 ± 2,03 | 0,52 |

List of abbreviations: TIR, Time in Range 70-140 mg/dL; TITR, Time in Tight range 70-140 mg/dL; TAR, Time Above Range 181-250 mg/dL; TAR250, Time Above Range > 250 mg/dL; TBR, Time Below Range 54-69 mg/dL; TBR54, Time Below Range < 54 mg/dL AG, Average Glucose; SD, Standard Deviation; GMI, Glucose management Indicator; CV, Coefficient of Variation; TDI, Total Daily Insulin Requirement; CHO, carbohydrates

**Supplementary Table 2**. Metrics of all patients (N=91) at baseline (T0) and after 1 (T1) and 3 months (T3) from baseline. Data are expressed as mean ± SD. Bold = statistically significant. **≥18 yrs n=32**

|  | **T0** | **T1** | **P**  ***(T1 vs T0)*** | **T3** | **P**  ***(T3 vs T0)*** |
| --- | --- | --- | --- | --- | --- |
| **TIR%** | **69,47 ± 9,84** | **72,59 ± 8,34** | 0,06 | 74,19 ± 8,84 | **0,003** |
| **TITR%** | **44,41± 9,77** | **49,56 ± 10,25** | **0,01** | 49,00 ± 9,44 | **0,01** |
| **TAR%** | **22,44 ± 5,65** | **19,12 ± 6,31** | **0,005** | 18,84 ± 5,84 | **0,001** |
| **TAR250%** | **7,03 ± 6,43** | **6,16 ± 4,47** | 0,61 | 5,53 ± 4,44 | 0,15 |
| **TBR%** | **1,16 ± 1,19** | **1,78 ± 2,04** | **0,03** | 1,22 ± 1,29 | 1 |
| **TBR54%** | **0,22 ± 0,49** | **0,28 ± 0,52** | 0.48 | 0,22 ± 0,55 | 1 |
| **AG (mg/dl)** | **157,66 ± 17,02** | **151,31 ± 15,87** | **0,05** | 151,19 ± 13,34 | **0,02** |
| **SD (mg/dl)** | **53,41 ± 9,90** | **53,34 ± 9,72** | 0,98 | 51,37 ± 10,73 | 0,20 |
| **GMI (%)** | **7,06 ± 0,35** | **6,91 ± 0,38** | **0,03** | 6,92 ± 0,33 | **0,01** |
| **CV (%)** | **33,79 ± 3,93** | **35,17 ± 4,42** | 0,09 | 33,91 ± 5,14 | 0,83 |
| **TDI (U/kg/day)** | **0,77 ± 0,26** | **0,79± 0,28** | 0,29 | 0,75 ± 0,25 | 0,17 |
| **Total Bolus (U/kg/day)** | **0,43 ± 0,17** | **0,46 ± 0,19** | 0,16 | 0,44 ± 0,18 | 0.72 |
| **Auto Correction Bolus (U/kg/day)** | **0,14 ± 0,09** | **0,18 ± 0,09** | **0,001** | 0,17 ± 0,09 | **0,02** |
| **Meal Bolus (U/kg/day)** | **0,31 ± 0,15** | **0,28 ± 0,14** | 0,15 | 0,27 ± 0,11 | 0,15 |
| **Basal (U/kg/day)** | **0,38 ± 0,27** | **0,33 ± 0,10** | 0,42 | 0,31 ± 0,08 | **0,02** |
| **Meals (n/day)** | **3,69 ± 1,27** | **3,51 ± 1,41** | 0,37 | 3,43 ± 1,18 | 0,30 |
| **CHO (g/day)** | **184,61 ± 68,43** | **170,19 ± 74,28** | 0,07 | 166,77 ± 67,31 | 0,06 |
| **CHO/kg (g/Kg/day)** | **3,15 ± 1,45** | **2,86 ± 1,80** | 0,06 | 2,73 ± 1,43 | **0,01** |

List of abbreviations: TIR, Time in Range 70-140 mg/dL; TITR, Time in Tight range 70-140 mg/dL; TAR, Time Above Range 181-250 mg/dL; TAR250, Time Above Range > 250 mg/dL; TBR, Time Below Range 54-69 mg/dL; TBR54, Time Below Range < 54 mg/dL AG, Average Glucose; SD, Standard Deviation; GMI, Glucose management Indicator; CV, Coefficient of Variation; TDI, Total Daily Insulin Requirement; CHO, carbohydrates
